# Construction and evaluation of a SPECT-based radiomics nomogram for predicting the therapeutic response to radioactive iodine in patients with differentiated thyroid carcinoma

**DOI:** 10.1097/MD.0000000000047658

**Published:** 2026-02-13

**Authors:** Haiyang Yu, Yingnan Jia, Qian Wu, Peihui Liu

**Affiliations:** a Department of Radiology, Yanbian University Affiliated Hospital (Yanbian Hospital), Yanji, Jilin Province, China; b Department of Nuclear Medicine, Southeast University Affiliated Xuzhou Central Hospital, Xuzhou, Jiangsu Province, China; c Department of Neuro-Intervention, Huludao Central Hospital, Huludao, Liaoning Province, China.

**Keywords:** clinical prognosis, logistic regression analysis, nomogram model, radiomics, thyroid neoplasms

## Abstract

Differentiated thyroid carcinoma (DTC) frequently requires radioactive iodine therapy, yet accurately predicting short-term therapeutic response remains challenging. Radiomics offers a means to quantify intratumoral heterogeneity from routine imaging. We aimed to develop and internally validate a Single-photon emission computed tomography (SPECT)-based radiomics nomogram for predicting 6-month response after RAI in DTC. We conducted a single-center retrospective study of 420 patients with DTC. Post-therapy ^131^I SPECT/CT images were processed to extract image biomarker standardisation initiative-aligned radiomic features in MATLAB; features passing reproducibility (ICC ≥ 0.80) and redundancy (|*r*|<0.85) filters were entered into a nested-cross-validation (CV) least absolute shrinkage and selection operator to derive a radiomics score, which was combined with clinicopathologic variables to build a nomogram. Model performance was assessed for discrimination (receiver operating characteristic area under the curve [AUC]), calibration (concordance index [C-index]; calibration intercept/slope), and clinical utility (decision-curve analysis); internal validation used outer 10-fold CV and 1000 bootstrap with permutation testing. Multicollinearity was checked by variance inflation factor. Interobserver reproducibility was high: 79/107 (73.8%) features achieved ICC ≥ 0.80 (median ICC 0.88), and the median mask-level Dice was 0.87. The nomogram showed AUC = 0.853 (95% confidence interval: 0.786–0.892) and C-index = 0.889 (95% confidence interval: 0.812–0.934) with good calibration. Internal validation yielded CV–AUC = 0.846 (SD 0.037) and bootstrap optimism-corrected C-index = 0.876/AUC = 0.848; permutation AUCs centered near 0.50. Decision-curve analysis indicated clinical net benefit across thresholds 0.15 to 0.95. All validation was internal; no external cohort was available. Radiomics score, age, regional lymph node metastasis, extrathyroidal extension, and tumor diameter independently influence the therapeutic outcome of RAI in DTC patients. In this exploratory, single-center study, the SPECT-based radiomics nomogram demonstrates promising internal performance; however, it must undergo external, multi-center validation and, if necessary, local recalibration before it can be considered for routine clinical use.

## 1. Introduction

According to epidemiological surveys conducted in 2020, thyroid carcinoma has become the seventh most common cancer in China, and its global incidence and mortality continue to rise annually; differentiated thyroid carcinoma (DTC) accounts for the vast majority of these cases.^[1,2]^ Radioactive-iodine (RAI) therapy is now widely used in the management of DTC, yet accurately predicting therapeutic response remains a major clinical challenge.^[3,4]^ Single-photon emission computed tomography (SPECT) is routinely employed during the diagnostic and therapeutic workflow for thyroid cancer, and mounting evidence indicates that SPECT plays an increasingly important role in both settings.^[5,6]^ In recent years, advances in image-acquisition techniques and standardized post-processing have driven rapid progress in radiomics. Radiomics extracts texture features, shape descriptors, and other quantitative parameters-often imperceptible to the naked eye-through fully or semi-automated software, enabling clinicians to identify and quantify imaging biomarkers that can aid in prognostic stratification for oncology patients.^[7,8]^ In parallel, predictive models-particularly nomograms-have gained widespread acceptance for assessing clinical outcomes in oncology, offering an intuitive and practical means of visualizing individualized risk.^[9,10]^ To evaluate the utility of a SPECT-based radiomics nomogram for predicting the efficacy of RAI therapy in patients with DTC, we conducted the present study, and the results are reported below.

## 2. Materials and methods

### 2.1. Study subjects

From January 2023 to December 2024, we retrospectively screened 512 consecutive patients with DTC who had undergone total or near-total thyroidectomy and were considered for postoperative RAI in our center. Among these, 12 patients were excluded because they did not undergo baseline post-therapy SPECT/CT (n = 7) or the SPECT/CT images were incomplete or of insufficient quality for radiomics analysis (n = 5). The remaining 500 patients underwent baseline post-therapy SPECT/CT and had available imaging data for radiomics analysis.

**Figure 1.**
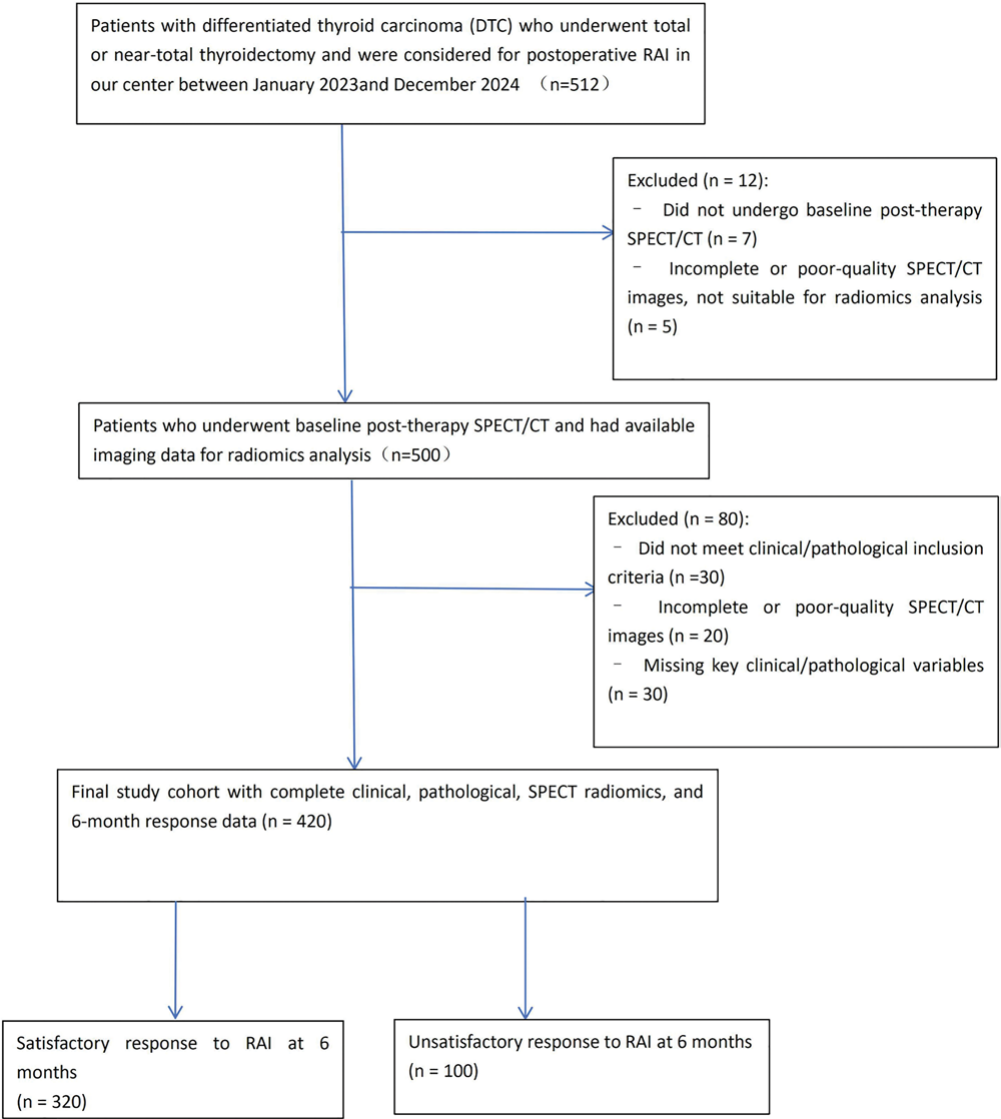
Flowchart of patient enrollment in the present study.

We then excluded a further 80 patients who did not meet the predefined clinical/pathological inclusion criteria (n = 30), had incomplete or poor-quality SPECT/CT images after quality control (n = 20), or had missing key clinical/pathological variables required for model development (n = 30). Finally, 420 patients with complete clinical, pathological, SPECT radiomics, and 6-month response data were included in the present analysis. According to the 6-month response to RAI, patients were categorized into a satisfactory-response group (n = 320) and an unsatisfactory-response group (n = 100). The patient selection process is summarized in the STROBE-compliant flow diagram (Fig. 1).

Inclusion criteria: all patients had histopathologically confirmed DTC according to established standards^[10]^; no concurrent malignancies; complete clinical and follow-up data available; and informed consent signed by all participants.

Exclusion criteria: patients with severe hepatic or renal dysfunction; patients who had undergone other major treatments (such as chemotherapy or radiotherapy) within 3 months prior to enrollment.

This study was approved by the Ethics Committee of our hospital (Approval No. LLWYH-2022-11-003). Written informed consent was obtained from all patients prior to participation and data collection.

### 2.2. Methods

Clinical data of patients were collected and recorded, and all patients were followed up for 6 months. Follow-up was conducted by trained specialists via telephone, outpatient visits, and WeChat communications. Patients were divided into 2 groups based on treatment outcomes at 6 months: a satisfactory-responsegroup (n = 320) and an unsatisfactory-response group (n = 100). Radiomics texture features from SPECT images were extracted using MATLAB, and a radiomics score (RS) was calculated for each patient. Multivariate logistic regression analysis identified independent risk factors influencing the therapeutic response to radioactive-iodine treatment in patients with DTC. Based on these independent factors, a nomogram was constructed. The predictive performance of the nomogram was validated in 3 ways: discrimination capability was evaluated using the receiver operating characteristic (ROC) curve; calibration was assessed by a calibration curve to measure the agreement between predicted and actual outcomes; clinical utility was evaluated through decision-curve analysis (DCA).

### 2.3. Outcomes/follow-up window

We selected a 6-month endpoint as an early-response time point aligned with major guidelines and routine practice in DTC. The 2015 American Thyroid Association (ATA) guideline recommends dynamic risk re-assessment at 6 to 12 months after initial therapy using serum thyroglobulin (Tg), neck ultrasound and, when appropriate, radioiodine imaging; this early window informs clinical decisions on surveillance and the need for additional therapy before longer-term outcomes are available. Consistent with this, contemporary DTC cohorts often schedule biochemical/functional reevaluation every 6 months in the first year following thyroidectomy and RAI. Therefore, a 6-month horizon is clinically meaningful for an imaging-based prediction model aimed at early stratification, while acknowledging that late recurrences can occur and require longer follow-up.

### 2.4. SPECT imaging protocol

Post-therapy 131I imaging was performed on a hybrid SPECT/CT system (Symbia Intevo 6; Siemens Healthineers, Erlangen, Germany) at Xuzhou Central Hospital. After oral administration of therapeutic 131I, planar whole-body scintigraphy was acquired according to our institutional routine, followed by SPECT/CT of the neck (and upper mediastinum when indicated) for anatomical localization. A high-energy parallel-hole collimator was used with a 364-keV photopeak and a 15% to 20% energy window. Images were reconstructed using the vendor-provided iterative algorithm with CT-based attenuation correction.

### 2.5. Image processing and extraction of texture features

To ensure cross-patient comparability before feature extraction, each 3D VOI was resampled to an isotropic voxel size of 2.0 × 2.0 × 2.0 mm^3^ using a third-order B-spline interpolation. Within each VOI, voxel intensities were standardized by *z*-score normalization (subtracting the mean and dividing by the standard deviation), and gray-level discretization was performed with a fixed bin width of 25 counts. Small morphological holes (<10 voxels) in the VOI were filled to avoid artificial fragmentation, and no additional smoothing or filtering was applied. Radiomic texture features were then calculated in MATLAB based on image biomarker standardisation initiative (IBSI)-compliant first-order, gray-level co-occurrence matrix, gray-level run-length matrix (GLRLM), gray-level size-zone matrix (GLSZM), GLDM, and neighborhood gray-tone difference matrix (NGTDM) formulations; the most informative features selected by least absolute shrinkage and selection operator (LASSO) are described in radiomics texture features selected by LASSO regression.

### 2.6. Radiomics feature extraction and selection

Rationale and software: We implemented the pipeline in MATLAB R2023a (Image Processing Toolbox v11.7; Statistics and Machine Learning Toolbox v12.5) to align with our PACS-side preprocessing/QA scripts and to ensure deterministic operators (version control and fixed random seeds). A 20-patient cross-check against PyRadiomics 3.0 showed high agreement for canonical features (median Spearman ρ > 0.95). All feature definitions followed the IBSI guidelines, and the key feature families and preprocessing parameters (resampling, discretization, normalization) are described in the following subsections to facilitate cross-software implementation. The MATLAB scripts used for image preprocessing and feature extraction are available from the corresponding author on reasonable request to support independent replication or implementation in open-source environments.

IBSI-aligned feature set: Following IBSI, we calculated 107 candidates: first-order (18), shape (14), GLCM (24), GLRLM (16), GLSZM (16), GLDM (14), NGTDM (5). Given SPECT SNR and dimensionality control, wavelet/LoG-derived features were piloted but not retained owing to instability/redundancy.

Preprocessing (standardization across patients): Before feature extraction, all volumes of interest were resampled to 2.0 × 2.0 × 2.0 mm^3^ (third-order B-spline); intensities underwent within-VOI *z*-score normalization; gray-level discretization used a fixed bin width of 25 counts. Morphological holes <10 voxels were filled; no additional smoothing was applied. These steps were applied identically to all patients to standardize voxel geometry and intensity scale across scans random seed = 2,02,40,101.

Reproducibility checks: On a random 15% (n = 60) subset with duplicate segmentations by 2 readers, we computed ICC (2, 1) with 95% confidence intervals (CIs; 1000 bootstrap) for each feature and Dice for masks. Features with ICC ≥ 0.80 were retained; others were excluded.

Feature reduction and selection: Steps: removal of near-zero variance features; redundancy filter based on pairwise Pearson correlation (|*r*| ≥ 0.85, retaining the feature with higher univariable area under the curve [AUC] and/or lower variance inflation factors); *z*-score standardization (mean 0, SD 1) within each training fold; and nested cross-validated LASSO-logistic regression for feature selection (outer 10-fold, inner 5-fold). In the inner loop, the LASSO penalty *λ* was tuned over a logarithmic grid using binomial deviance as the objective function. We applied the 1 − SE rule and chose the largest *λ* whose deviance lay within one standard error of the minimum to favor a parsimonious solution. Around the selected *λ*, the deviance curve formed a broad plateau and the coefficient paths were stable, with 4 radiomic features retaining non-zero coefficients over a range of neighboring *λ* values. A RS was then calculated as a weighted sum of the selected standardized features and subsequently combined with clinicopathologic covariates in the multivariable nomogram, with optimism correction via bootstrap resampling.

### 2.7. Observation indicators and evaluation methods

General clinical data at admission, including age, sex, body mass index, and tumor-related parameters such as tumor diameter, tumor-node-metastasis (TNM) stage, and the presence or absence of regional LN metastasis, were recorded. Therapeutic outcomes were evaluated according to the 2015 ATA guidelines for DTC.^[11]^ Treatment response was assessed using a composite of postoperative serum Tg levels and the post-therapy whole-body radioactive iodine scan (Rx-WBS) performed after ^131^I administration. A satisfactory therapeutic response was defined as a negative Rx-WBS and a Tg level < 1 mg/L in the absence of interfering anti-thyroglobulin antibody (TgAb), whereas an unsatisfactory response was defined as a positive Rx-WBS and/or a Tg level ≥ 1 mg/L. When TgAb was known to be positive or rising, Tg values were interpreted with caution and Rx-WBS findings took precedence for response classification rather than relying on Tg alone. Because TgAb was not systematically available for all patients, some degree of endpoint misclassification cannot be completely excluded and is acknowledged as a limitation. Potential confounders with established prognostic relevance in DTC, such as BRAF V600E and TERT promoter mutations and TgAb-were not routinely measured at our institution during the study period and were missing in most eligible patients. In line with our prespecified missing-data policy, variables with more than 50% missingness were excluded from model building to avoid unstable estimates and biased regression coefficients. Consequently, systematic adjustment for these markers was not feasible in the present analysis, and residual confounding by molecular and serologic factors (including BRAF, TERT and TgAb) cannot be excluded. TgAb status was available in a subset of patients; overall, 59 of 420 patients (14.0%) were TgAb-positive, which is slightly lower than the ~20% to 30% TgAb positivity reported in other postoperative DTC cohorts.

### 2.8. ROI segmentation and reproducibility

Two board-certified nuclear medicine physicians (reader A, 10 years of experience; reader B, 5 years) independently delineated 3-dimensional volumes of interest on post-therapy 131I SPECT/CT images while blinded to clinical outcomes. After a brief calibration session, both readers re-segmented a random 15% subset (n = 60) to assess interobserver variability. Radiomic features were extracted from each mask using identical preprocessing (isotropic resampling to 2.0 mm^3^, fixed bin width of 25 for intensity discretization, and *z*-score normalization). Interobserver feature reproducibility was quantified using a 2-way random-effects,absolute-agreement, single-measure intraclass correlation coefficient (ICC [2,1]), with 95% CIs derived from 1000 bootstrap resamples; features with ICC ≥ 0.80 were retained. To complement feature-level ICCs, we also computed Dice similarity coefficients between paired masks. Intraobserver repeatability was assessed in 30 cases re-segmented by reader A after an interval of at least 2 weeks, analyzed using ICC (3,1).

### 2.9. Statistical analysis

Statistical analyses were performed using R software (version 3.6.1; R Foundation for Statistical Computing, Vienna, Austria) and MATLAB R2023a (MathWorks, Natick). Continuous variables are reported as mean ± standard deviation or median (interquartile range), and categorical variables as counts (%). Group comparisons used the *t*-test or Mann–Whitney *U* test for continuous variables and the χ^2^ or Fisher’s exact test for categorical variables, as appropriate.

To construct the RS, we fitted a LASSO-logistic regression model to the standardized radiomic features in a nested CV framework, using binomial deviance to tune the penalty parameter λ and applying the 1 − SE rule to favor a parsimonious solution. The RS was then combined with clinical and tumor-related variables in a multivariable logistic regression model to build the nomogram. Model performance was evaluated in terms of discrimination (area under curve [AUC] and concordance index [C-index]), calibration (calibration plot, intercept and slope), and clinical usefulness [DCA]. Internal validation relied on stratified 10-fold CV and 1000 bootstrap resampling to obtain optimism-corrected estimates of AUC/C-index and calibration.

For continuous predictors (RS, age, tumor diameter), we explored potential non-linear relationships with the log-odds of unsatisfactory response using restricted cubic splines; because the curves were approximately linear over the observed ranges, the final model used simple linear terms. Multicollinearity was assessed using variance inflation factors, with variance inflation factors ≥ 5 predefined as indicative of problematic collinearity. All tests were 2-sided and *P* < .05 was considered statistically significant. More detailed technical settings (e.g. tuning grids and random seeds) are available from the corresponding author on reasonable request.

## 3. Results

### 3.1. Radiomics texture features selected by LASSO regression

The radiomic features ultimately selected by LASSO regression were as follows: coarseness (CRS), derived from the NGTDM, which quantifies gray-level differences between neighboring voxels; histogram variance (Hist), a first-order descriptor of intensity distribution within the volume of interest; short-run low gray-level emphasis (SRLGE), calculated from the GLRLM and characterizing the frequency of short runs with low intensity; and zone-size variance (ZSV), obtained from the GLSZM and capturing variability in the size of homogeneous intensity zones. In the nested CV procedure, the inner 5-fold curves of deviance and AUC showed a broad plateau around the selected penalty, and the 1 − SE rule consistently identified a λ value that retained these 4 non-zero radiomic features while avoiding overfitting.

### 3.2. Construction of radiomics score (RS)

The RS for each patient was calculated using the following linear model, which corresponds to the final LASSO coefficients: RS = −8.456 × 10^−6^ × Hist − 3.332 × SRLGE + 6.789 × 10^−4^ × ZSV − 57.894 × CRS. This formula specifies the 4 selected radiomic features and their weights and allows the RS to be reproduced in external datasets that apply the same preprocessing and standardization.

### 3.3. Interobserver reproducibility

Across 107 extracted features, 79 (73.8%) achieved ICC ≥ 0.80 (median ICC 0.88, IQR 0.83–0.92); 42 features showed excellent reproducibility (ICC ≥ 0.90). The median Dice between reader masks was 0.87 (IQR 0.84–0.90). Intraobserver repeatability (reader-A)was similarly high (median ICC 0.90). Only ICC-qualified features were carried forward to feature selection and model development (see methods radiomics feature extraction and selection).

### 3.4. Comparison of general clinical data

Baseline characteristics differed significantly between groups with respect to age and sex (Table 1).

**Table 1:** Comparison of general baseline characteristics between the 2 groups of differentiated thyroid carcinoma patients.

Variable	Satisfactory group (n = 320)	Unsatisfactory group (n = 100)	χ^2^/*t* value	*P* value
Gender (n)			5.498	.019
Male	285	80		
Female	35	20		
BMI/(kg/m^2^), x ± *s*	22.67 ± 3.87	22.56 ± 3.83	0.249	.402
Age (yr), x ± *s*	60.78 ± 6.43	65.87 ± 5.89	−7.045	5.86 × 10^−12^
Education level (n)			0.134	.715
Junior high school or below	189	57		
High school or above	131	43		
History of hypertension (n)	165	54	0.181	.670
History of diabetes (n)	78	23	0.079	.779
History of coronary disease (n)	72	20	0.278	.598
History of stroke (n)	30	11	0.228	.633

Values are mean ± SD or n (%), as appropriate.

*P* values for continuous variables are from Welch’s *t* test.

*P* values for categorical variables are from Pearson’s χ^2^ test (2-sided).

BMI = body mass index, DTC = differentiated thyroid carcinoma, RAI = radioactive iodine, SD = standard deviation.

### 3.5. Comparison of tumor-related characteristics between groups

Comparison of tumor-related characteristics showed statistically significant differences between the satisfactory and unsatisfactory response groups in terms of tumor diameter, extrathyroidal extension (ETE), TNM stage, regional LN metastasis, ATA risk stratification, and RS score (all *P* < .001), as shown in Table 2.

**Table 2:** Comparison of tumor-related characteristics between the 2 groups of patients with differentiated thyroid carcinoma.

Variable	Satisfactory group (n = 320)	Unsatisfactory group (n = 100)	χ^2^/*t* value	*P* value
Tumor location (n)				
Unilateral	245	75	0.103	.748
Bilateral	75	25		
Tumor diameter (cm), x ± *s*	2.89 ± 1.23	3.89 ± 1.67	−6.479	1.56 × 10^−7^
Multifocality (n)				
Absent	156	53	0.550	.458
Present	164	47		
Extrathyroidal extension (n)				
Absent	140	65	13.770	2.07 × 10^−4^
Present	180	35		
TNM stage (n)				
Stage I	230	49	20.14	4.23 × 10^−5^
Stage II	90	50		
Stage III	0	1		
Regional lymph-node metastasis (n)				
Yes	131	65	17.725	2.55 × 10^−5^
No	189	35		
ATA risk stratification (n)				
Low risk	170	45	26.811	6.50 × 10^−5^
Intermediate risk	140	40		
High risk	10	15		
RS score, x ± *s*	0.56 ± 0.21	1.19 ± 0.28	−24.063	6.55 × 10^−44^

Tests as in Table 1.

ATA = American Thyroid Association, DTC = differentiated thyroid carcinoma, ETE = extrathyroidal extension, LN = lymph node, RAI = radioactive iodine, RS = radiomics score.

### 3.6. Multivariate logistic regression analysis of prognostic factors affecting the therapeutic efficacy of radioactive iodine in patients with differentiated thyroid carcinoma

A multivariate logistic regression analysis was conducted to identify independent prognostic factors influencing the efficacy of radioactive iodine (RAI) therapy in patients with DTC. The treatment response (unsatisfactory = 1, satisfactory = 0) was used as the dependent variable, while independent variables were selected based on those that showed statistically significant differences in baseline clinical and tumor-related data. These included: age (entered as a continuous variable), sex (male = 1, female = 0), tumor diameter (cm), regional LN metastasis (yes = 1, no = 0), ETE (yes = 1, no = 0), TNM stage (stage I = 1, stage II = 2, stage III = 3), ATA risk stratification (low risk = 1, intermediate risk = 2, high risk = 3), and (RS, entered as a continuous variable). The results revealed that the independent risk factors associated with poor therapeutic response included RS (odds ratio [OR] = 4.685, 95% CI: 3.294–5.193), age (OR = 2.634, 95% CI: 1.973–3.016), regional LN metastasis (OR = 3.967, 95% CI: 2.149–4.637), ETE (OR = 4.112, 95% CI: 3.251–5.682), and tumor diameter (OR = 7.538, 95% CI: 6.594–8.125), as shown in Table 3. Collinearity check and scaling. As expected, tumor diameter, regional LN metastasis and ETE are clinically related markers of tumor burden and invasiveness. However, variance inflation factors for the final predictors were RS 1.74, age 1.36, tumor diameter 2.12, regional LN metastasis 1.58, and ETE 1.43-all <3, suggesting that this correlation did not materially inflate standard errors or destabilize coefficient estimates. Continuous variables were entered on interpretable scales (RS as *z*-score; tumor diameter per 1-cm increase), so the relatively large OR for diameter reflects a steep risk gradient rather than collinearity. In this context, “independent” refers to variables that remained significantly associated with treatment response after mutual adjustment, while representing partially overlapping yet complementary dimensions of tumor burden and invasiveness. In additional exploratory models using restricted cubic splines and categorized tumor diameter, the predicted probability of unsatisfactory response increased monotonically with size and did not show a marked threshold or reversal, indicating that the large per-centimeter OR in Table 3 summarizes a stable, approximately linear risk gradient across the observed diameter range.

**Table 3:** Multivariable logistic regression analysis of prognostic factors affecting the therapeutic efficacy of radioactive iodine in patients with differentiated thyroid carcinoma.

Factor	β Coefficient	SE	Wald χ^2^	*P* value	OR (95% CI)	VIF
Radiomics score (RS)	1.544	0.116	176.87	2.34 × 10^−40^	4.685 (3.294–5.193)	1.74
Age	0.969	0.108	80.03	3.68 × 10^−19^	2.634 (1.973–3.016)	1.36
Regional LN metastasis	1.378	0.196	49.34	2.16 × 10^−12^	3.967 (2.149–4.637)	1.58
Extrathyroidal extension (ETE)	1.414	0.142	98.54	3.18 × 10^−23^	4.112 (3.251–5.682)	1.43
Tumor diameter (per 1-cm increase)	2.020	0.053	1438.32	9.90 × 10^−315^	7.538 (6.594–8.125)	2.12

OR per stated scale; age is expressed in years; tumor diameter is measured per 1-cm increase.

CI = confidence interval, ETE = extrathyroidal extension, LN = lymph-node, OR = odds ratio, RS = radiomics score, SE = standard error, VIF = variance inflation factors.

### 3.7. Construction of a radiomics-based nomogram model

**Figure 2.**
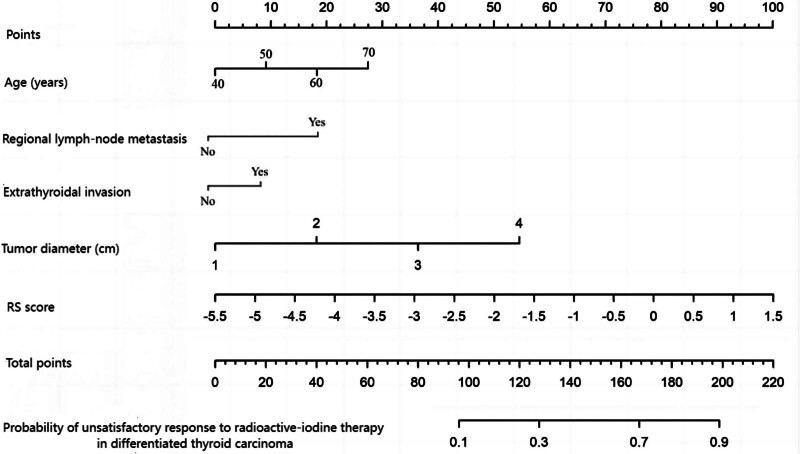
Nomogram model based on SPECT radiomics for predicting the therapeutic efficacy of radioactive iodine in patients with differentiated thyroid carcinoma. SPECT = single-photon emission computed tomography.

A nomogram model predicting the therapeutic efficacy of RAI in patients with DTC was constructed using the “rms” package in R, based on SPECT radiomics features. In this study, treatment outcomes were presented in the form of the RS. The nomogram integrates multiple independent risk factors-including RS, age, regional LN metastasis, ETE, and tumor diameter-to estimate the probability of an unsatisfactory response. By mapping an individual patient’s values for each risk factor onto the nomogram, clinicians can intuitively estimate the likelihood of an unsatisfactory treatment response, as shown in Figure 2.

### 3.8. Validation of the predictive performance of the nomogram model

**Figure 3.**
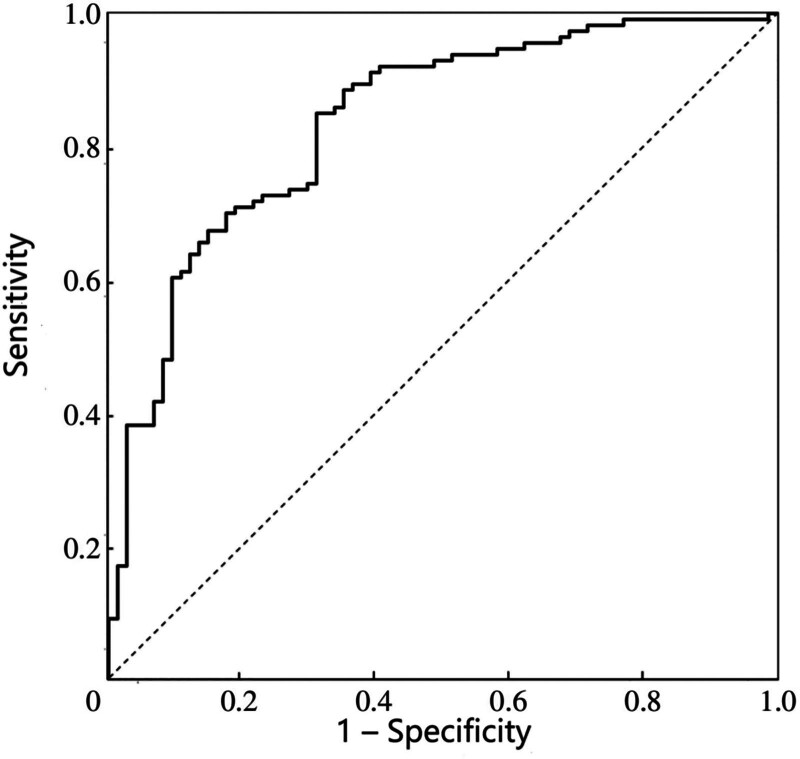
ROC curve validating the predictive performance of the constructed nomogram model. ROC = receiver operating characteristic.

The predictive performance of the constructed nomogram model was validated using ROC curve analysis. The results showed that the area under the curve (AUC) was 0.853 (95% CI: 0.786–0.892), indicating that the nomogram model had good predictive accuracy, as shown in Figure 3.

### 3.9. Evaluation of the accuracy of the nomogram model

**Figure 4.**
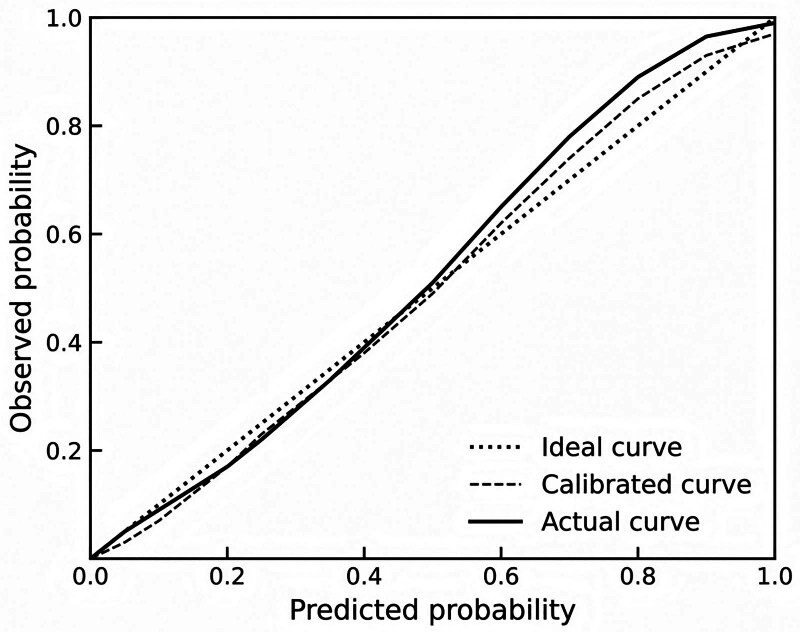
Calibration curve based on bootstrap validation of the predictive accuracy of the constructed nomogram model.

The results showed that the C-index of the nomogram model was 0.889, and the 95% CI obtained via bootstrap resampling was 0.812 to 0.934, indicating that the model had good predictive accuracy. A calibration curve generated using the bootstrap method further demonstrated good agreement between predicted and observed outcomes, reflecting a high degree of model calibration, as shown in Figure 4.

### 3.10. Internal validation and overfitting checks

Internal validation and overfitting checks. In outer 10-fold cross-validation (CV), the nomogram achieved a mean CV-AUC of 0.846 (SD 0.037) with a Brier score of 0.142. Calibration was satisfactory (intercept −0.02, slope 0.98), indicating minimal optimism. Bootstrap resampling (1000 iterations) yielded an optimism-corrected C-index of 0.876 (95% CI: 0.842–0.905) and an optimism-corrected AUC of 0.848, consistent with the apparent AUC reported above (0.853). In permutation tests (2000 iterations), the median AUC was 0.501 (IQR 0.492–0.509), and no permuted AUC exceeded the observed AUC (empirical *P* < .001), further arguing against overfitting. All performance metrics reported above reflect internal validation (nested CV and bootstrap); no independent external validation cohort was available in this retrospective single-center study, and the current nomogram should therefore be regarded as internally validated only.

### 3.11. Clinical utility of the nomogram model

**Figure 5.**
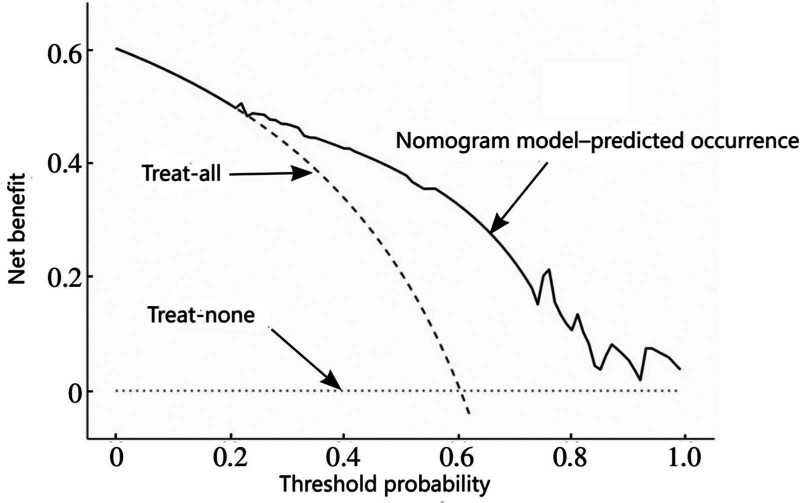
Decision curve analysis (DCA) of the constructed nomogram model. DCA = decision curve analysis.

The DCA showed that the applicable threshold probability range was 0.15 to 0.95, indicating substantial clinical net benefit of the nomogram model, as shown in Figure 5.

### 3.12. Sensitivity analysis excluding TgAb-positive patients

Given the potential for TgAb to interfere with Tg-based response classification, we repeated the multivariable logistic regression after excluding all TgAb-positive patients (59/420, 14.0%), leaving 361 patients in the sensitivity cohort. The same set of predictors-RS, age, tumor diameter, regional LN metastasis, and ETE-were retained in the model, and their effect estimates were broadly similar to those in the primary analysis (Table S1, Supplemental Digital Content, https://links.lww.com/MD/R366). Overall model discrimination and calibration were also comparable to the full-cohort nomogram, suggesting that TgAb-related misclassification is unlikely to fully explain the observed radiomics associations. These findings are summarized and interpreted in the following discussion.

## 4. Discussion

### 4.1. Principal findings and comparison with prior evidence

In this single-center cohort of 420 patients with DTC, we found that a SPECT-based radiomics nomogram combining a 4-feature RS with age, tumor diameter, regional LN metastasis and ETE achieved good internal discrimination and calibration (AUC 0.85; optimism-corrected AUC 0.85) for predicting 6-month response to RAI. DTC accounts for ~95% of all thyroid cancer cases. Although the 5-year survival rate for patients with DTC exceeds 98%, a subset of patients experience postoperative recurrence or persistent disease, necessitating RAI therapy. Among patients undergoing RAI therapy, approximately one-third require multiple treatment sessions to achieve disease control.^[12,13]^ In clinical practice, RAI has become a cornerstone of treatment for patients with DTC.

Currently, the therapeutic response to RAI is typically evaluated using Rx-WBS in combination with relevant serological markers.^[14,15]^ Prognostic assessment in oncology generally relies on the TNM staging system and recurrence risk stratification. However, studies have indicated that these conventional approaches are suboptimal for predicting the treatment outcomes of RAI in patients with DTC.^[16,17]^

In clinical practice, with the continuous advancement of imaging technology and computational big data analysis, radiomics-based on preoperative imaging features and integrated with disease prediction models-has been widely applied to predict tumor prognosis, yielding favorable clinical outcomes. Radiomics refers to the process of extracting imaging data from original medical images using specialized software, converting these data into quantifiable imaging features, and subsequently analyzing them using advanced algorithms. The analytical results are then combined with disease-specific characteristics to support clinical decision-making.

According to the radiomics hypothesis, heterogeneity in gene expression leads to intratumoral heterogeneity. Radiomics enables the extraction of imaging features that can reflect this heterogeneity more accurately than visual assessment alone. Over the past 3 years, an increasing number of studies have demonstrated that radiomics can effectively characterize tissue properties, accurately predict therapeutic responses, and assess patient prognosis. Notably, radiomics-basedparameters have been shown to outperform standardized uptake value-based metrics in evaluating treatment response and clinical outcomes in cancer patients.^[18,19]^ In this study, we used SPECT imaging as the source for radiomics feature extraction. SPECT has demonstrated high diagnostic accuracy in evaluating DTC, particularly in the context of RAI therapy, where it enables precise identification and characterization of cervical LN metastases. Therefore, SPECT-based radiomics may hold substantial clinical relevance for assessing RAI response in patients with DTC.^[20,21]^ LASSO regression was used to select meaningful texture features, including coarseness (derived from the NGTDM), histogram variance, short-run low grayscale enhancement (from the GLRLM), and ZSV (from the GLSZM). These 4 features may reflect the underlying genetic heterogeneity of DTC, providing important insights into tumor biology that are closely associated with treatment outcomes. A RS was then calculated for each patient using a linear fitting model, enabling a quantitative evaluation of radiomics-based risk in individual patients.

### 4.2. Biologic plausibility of radiomic features

This study identified several independent risk factors affecting the therapeutic response to RAI in patients with DTC through multivariate logistic regression analysis. These included the RS, age, regional LN metastasis, ETE, and tumor diameter. Biological plausibility of the predictors. Beyond statistical significance, the direction and composition of the RS are biologically coherent with radioiodine pharmacodynamics. Among the selected features, (ZSV; GLSZM) carried a positive coefficient in the RS, indicating that a mixture of very small and very large iodine-avid zones (i.e., high structural heterogeneity) is associated with poorer RAI response-consistent with dedifferentiation, patchy NIS expression, and uneven intratumoral dose deposition. In contrast (CRS; NGTDM) showed a negative coefficient, implying that low spatial-frequency, more uniform uptake patterns track with better response, in line with well-differentiated tissue that retains organized iodide trapping/organification. SRLGE and GLRLM also entered the RS with a negative weight; in our reconstruction and discretization setting, higher SRLGE reflects fine but limited low-uptake runs embedded in otherwise uniform uptake, which likely does not disrupt global iodine delivery. Histogram variance contributed only marginally (small negative weight), suggesting that localized texture heterogeneity (ZSV) is more informative for RAI effectiveness than global intensity spread in this cohort. Together, these patterns support the notion that spatially coherent, homogeneous iodine uptake favors effective dose accumulation, whereas mosaic uptake architectures signal reduced biological avidity and worse response.^[22,23]^ From a pathophysiological standpoint, the heterogeneity measures derived from GLSZM (e.g., ZSV) and NGTDM (e.g., coarseness) may serve as imaging surrogates of progressive dedifferentiation and loss of iodine avidity. In well-differentiated DTC, NIS expression and iodide organification are relatively uniform across tumor cells, giving rise to large, homogeneous iodine-avid zones and low ZSV values. As clonal dedifferentiation develops, NIS expression becomes patchy and is downregulated in subsets of cells, producing a mixture of intensely avid foci interspersed with iodine-poor regions; this fragmented uptake architecture is captured as higher ZSV and altered NGTDM patterns. Experimental and clinical data indicate that such spatially heterogeneous NIS expression is a hallmark of radioiodine-refractorydisease, linking these radiomics heterogeneity features to both reduced functional iodine trapping and histological progression towards less differentiated phenotypes. Beyond these qualitative interpretations, the texture families contributing to the RS are consistent with emerging radiogenomic and microenvironmental data in thyroid cancer. Recent studies integrating CT-, US-, or SPECT-derived radiomics with genomic profiling have reported that heterogeneity-related features from GLSZM, GLRLM and NGTDM families-similar to our ZSV, SRLGE and coarseness-tend to be enriched in tumors harboring BRAF V600E and/or TERT promoter mutations, in tall-cell or poorly differentiated histotypes, and in gene-expression signatures of MAPK pathway activation and cell-cycle proliferation. These adverse molecular profiles are, in turn, associated with reduced sodium-iodide symporter (NIS) expression, fibro-inflammatory stromal remodeling, and an “immunologically altered” tumor microenvironment that favor radioiodine refractoriness. In addition, heterogeneity-type radiomic descriptors have been linked to increased stromal and immune-cell infiltration on histology and transcriptomics, suggesting that SPECT texture patterns may partly encode complex interactions between tumor epithelium, vasculature, and the surrounding microenvironment rather than simply reflecting tumor size or shape. Linking clinical covariates to iodine biology. The associations of age, regional lymph node (LN) metastasis, ETE, and tumor diameter with unsatisfactory response are also mechanistically plausible in the context of radioiodine biology. Advancing age is linked to progressive dedifferentiation and diminished NIS function, reducing iodide trapping and organification. LN metastasis and ETE reflect greater tumor burden and invasive phenotype, often accompanied by stromal remodeling, hypoxia, and heterogeneous NIS expression, all of which can impair uniform iodine uptake. Larger tumors are prone to necrosis/fibrosis and perfusion gradients, creating iodine-poor subregions that dilute the effective absorbed dose despite adequate macroscopic delivery. Consistent with this biology, our model showed a steep, approximately linear increase in the odds of unsatisfactory response with increasing tumor diameter, which explains the seemingly large per-centimeter OR and argues against this effect being a mere artifact of model instability. Notably, our RS integrates these microscopic texture cues, and its independent effect alongside age/LN/ETE/diameter suggests that meso-/micro-scale uptake architecture captured by radiomics adds complementary biological information beyond conventional morphology.^[24]^ Taken together, these diagnostics suggest that the seemingly large per-centimeter OR for tumor diameter reflects a steep but stable and approximately linear risk gradient across the observed size range, rather than numerical instability or artefactual amplification due to multicollinearity.

### 4.3. Model performance and potential clinical applicability

Based on the independent risk factors identified through multivariate logistic regression analysis, our team developed a nomogram model to predict the therapeutic response to RAI in patients with DTC. Validation of the nomogram demonstrated promising internal predictive performance, as indicated by a relatively high area under the ROC curve and good apparent calibration. DCA suggested potential clinical net benefit across a broad range of threshold probabilities under the assumptions of our retrospective cohort. However, these performance estimates are based on internal validation only and should be interpreted as hypothesis-generating rather than definitive evidence of clinical utility. In summary, RS, age, regional LN metastasis, ETE and tumor diameter remained statistically significant after mutual adjustment and together form a parsimonious set of prognostic covariates for early RAI response in DTC patients.^[25]^

### 4.4. Methodological robustness, limitations, and future work

This study used a retrospective, single-center design, which inherently restricts the generalizability of our findings. The cohort was drawn entirely from one tertiary referral hospital and therefore reflects our local surgical pathways, RAI dosing protocols, and SPECT acquisition/reconstruction procedures. Despite the use of consecutive case inclusion and prespecified eligibility criteria, referral patterns and treatment practices at our institution may differ from those at other centers, introducing selection and spectrum bias. As a result, the current nomogram should be considered internally validated only, and external, multi-center studies with harmonized imaging protocols are required before the model can be safely extrapolated to other clinical settings. These steps substantially reduce, though cannot entirely eliminate, the risk of overfitting; hence our emphasis on external, multi-center validation in future work.^[26]^ Taken together, the single-center retrospective design, internal-only validation, and lack of long-term outcomes indicate that this work should be regarded as an exploratory modeling study. Consequently, the present model should be interpreted as a hypothesis-generating decision-supporttool rather than a ready-to-use clinical instrument, and we do not recommend direct implementation in other settings without prior external validation and local recalibration.

Potential clinical use (after external validation): After external validation and local calibration, the model output (predicted probability of an unsatisfactory early RAI response) could support risk-stratified decision-making. For example, reatment adjustment – patients at higher predicted risk (e.g., predicted probability ≥ 0.50, calibrated locally) may warrant discussion of dose optimization, redifferentiation strategies, or clinical trial options; follow-up intensity – patients at lower predicted risk (e.g., predicted probability ≤ 0.20) may undergo routine surveillance, potentially avoiding unnecessary testing. Decision thresholds should be institution-specific, guided by DCA, resource considerations, and updated when external validation data become available.^[27]^

A major limitation of this work is the absence of an external validation cohort; therefore, the model’s transportability beyond our center remains uncertain and requires external, multi-center validation. This study is retrospective and single-center, which inherently introduces several sources of bias. Moreover, because this was a retrospective study using all eligible patients within the study period, no formal a priori power or sample size calculation was performed. Instead, we constrained model complexity based on the observed events-per-variable ratio and used penalized regression with nested CV and bootstrap validation to mitigate overfitting. Nonetheless, future prospective studies should pre-specify target sample sizes and power to optimize model development and validation. In addition, class imbalance was present (unsatisfactory responders = 100 vs 320 satisfactory), which can widen uncertainty around effect sizes and calibration in the minority class. We used stratified nested CV and bootstrap optimism correction to mitigate this, but residual instability in minority-class estimates cannot be excluded; future studies with larger unsatisfactory cohorts are warranted. First, selection bias may arise because patient inclusion depended on the availability of complete clinical and imaging data and on local referral/treatment practices; although we used consecutive cases and prespecified eligibility criteria, unmeasured factors could still influence cohort composition. Second, information bias is possible given variability and occasional incompleteness in routine clinical records (e.g., laboratory results, comorbidities, adjuvant therapies). Some potentially relevant variables (such as certain molecular markers) were unavailable for a proportion of patients; consequently, residual confounding cannot be excluded. Third, despite internal standard operating procedures, variability in SPECT acquisition and reconstruction (e.g., scanner hardware, acquisition time per view, energy windows, attenuation/scatter correction, and reconstruction parameters) may have introduced feature heterogeneity that the radiomics pipeline cannot fully remove. In the present study, all images were acquired on a single Siemens Symbia Intevo 6 SPECT/CT system, so cross-scanner feature stability could not be empirically evaluated; external multi-center studies using different scanners, phantom-based quality assurance, and, where appropriate, feature harmonization methods (e.g., ComBat) will be needed to confirm reproducibility across platforms. We attempted to mitigate these issues through consecutive sampling, prespecified feature-engineering and model-building steps, and internal bootstrap validation; however, the retrospective design precludes control over exposure and outcome assessment, and causality cannot be inferred. Prospective, multi-center studies using harmonized imaging protocols and predefined data-collection templates, together with external validation and longer follow-up, are needed to confirm the robustness and generalizability of our findings.

Limitation related to the 6-month outcome window. Another important limitation is that we used the response status at 6 months after RAI as the primary endpoint. In current clinical practice, an initial structural/biochemical response is often assessed around 6 to 12 months as part of “early dynamic risk stratification” and to guide near-term management decisions. However, DTC may recur years after initial therapy, and a 6-month response window cannot fully capture durable treatment success or late recurrences. Therefore, the present nomogram should be interpreted as predicting early RAI response rather than long-term cure. In this retrospective cohort, follow-up beyond 6 months was heterogeneous and incomplete, so we were not able to reliably test whether early non-response independently predicts long-term outcomes. Future prospective studies with standardized, long-term follow-up (e.g., ≥3–5 years) are needed to evaluate the model’s performance for late recurrence and disease-specific survival.

An additional limitation is the unavailability of key molecular and serologic prognostic markers (e.g., BRAF V600E, TERT promoter mutations, TgAb) for most patients, reflecting routine practice during the study period rather than selective exclusion. In accordance with our predefined rule, variables with more than 50% missingness were not entered into the multivariable model. While this approach avoids highly unstable and potentially misleading regression coefficients, it also means that we could not fully adjust for molecular risk. In addition, our 6-month response endpoint relied on a composite of Tg and Rx-WBS. Although Rx-WBS findings were prioritized when TgAb was known or suspected to interfere with Tg measurement, TgAb was not systematically measured in all patients, so some degree of outcome misclassification is possible. As an additional robustness check, we repeated the multivariable analysis after excluding all TgAb-positive patients, and the resulting coefficients and overall model performance were similar to those of the primary nomogram (Table S1, Supplemental Digital Content, https://links.lww.com/MD/R366). Therefore, residual confounding by unmeasured molecular and serologic factors cannot be excluded, and future prospective cohorts will need to collect these markers systematically and assess their incremental value and potential impact on model coefficients. Looking ahead, integrative “radiogenomic” and radiomic-microenvironment studies that combine SPECT texture features with systematic assessment of BRAF/TERT status, NIS and proliferation markers, immune-cell and stromal signatures, and hypoxia/fibrosis surrogates will be essential to formally test the mechanistic hypotheses suggested by the present RS and to clarify which molecular and microenvironmental phenotypes underlie the radiomics-RAI response associations observed here.

Single-center design limits the generalizability of our findings. Patient spectrum, referral patterns, surgical/RAI protocols, and imaging workflows at our institution may differ from those at other centers, which can shift predictor distributions and baseline risk (“spectrum effects”). In addition, radiomics features are sensitive to acquisition/reconstruction settings; even with internal SOPs, scanner hardware and parameter choices may vary elsewhere and impact feature stability. Although we used consecutive sampling and internal bootstrap validation to mitigate optimism, external performance in different hospitals and scanners remains unknown. Therefore, our model should be considered internally validated only, pending external, multi-center validation and potential model updating (e.g., intercept/slope recalibration or re-estimation of coefficients) before clinical adoption.

Future work will prioritize external, multi-center validation across geographically and demographically diverse cohorts and different SPECT scanners/protocols.^[28]^ We plan a prospective registry with harmonized acquisition/reconstruction settings (and phantom-based QA), pre-registered analysis, and adherence to TRIPOD/RQS recommendations. We will assess transportability via temporal and geographic validation, apply feature harmonization where appropriate (e.g., ComBat), and perform model updating if calibration drift is observed (recalibration-in-the-large, slope adjustment, or revision of coefficients). Clinical utility will be evaluated with DCA and, where feasible, clinical impact assessments to determine whether the nomogram changes management or outcomes.

Although ICC-based filtering and mask-level dice similarity coefficients supported good reproducibility, radiomics features remain sensitive to segmentation nuances; thus, residual observer effects cannot be fully excluded and warrant standardization and external validation in future work. Finally, although our current radiomics implementation is MATLAB-based, all features are IBSI-compliant, key preprocessing parameters are fully reported, and cross-checking against PyRadiomics in a subset suggested high cross-software concordance; nevertheless, independent replication using open-source pipelines in external cohorts will be important to further strengthen reproducibility.

## 5. Conclusion

RS, age, regional LN metastasis, ETE, and tumor diameter were independently associated with 6-month RAI response. Our findings support the hypothesis that SPECT-based radiomics heterogeneity measures may capture early dedifferentiation and reduced iodine avidity; nevertheless, this mechanistic link remains hypothesis-generating and will require confirmation in future radiogenomic and microenvironmental studies. The SPECT-based radiomics nomogram demonstrates promising internal performance; however, it must undergo external, multi-center validation and, if necessary, local recalibration before it can be considered for routine clinical use.

## Author contributions

**Data curation:** Peihui Liu.

**Formal analysis:** Peihui Liu.

**Funding acquisition:** Peihui Liu.

**Investigation:** Peihui Liu.

**Methodology:** Haiyang Yu, Yingnan Jia, Peihui Liu.

**Project administration:** Haiyang Yu, Yingnan Jia, Peihui Liu.

**Resources:** Haiyang Yu, Yingnan Jia, Qian Wu.

**Software:** Yingnan Jia, Qian Wu.

**Supervision:** Yingnan Jia, Qian Wu.

**Validation:** Yingnan Jia, Qian Wu.

**Visualization:** Yingnan Jia.

**Writing – original draft:** Yingnan Jia.

**Writing – review & editing:** Haiyang Yu, Yingnan Jia.

## Supplementary Material


