# Supplementary material for: Construction and evaluation of a SPECT-based radiomics nomogram for predicting the therapeutic response to radioactive iodine in patients with differentiated thyroid carcinoma
Source: Medicine (Baltimore). 2026 Feb 13;105(7):e47658. doi: 10.1097/MD.0000000000047658 (PMC12908823; doi:10.1097/MD.0000000000047658)
Supplement: Supplementary file 1 [file medi-105-e47658-s001.docx]

# Supplementary Table S1. Multivariable logistic regression of unsatisfactory response to RAI: primary analysis (full cohort, n = 420) versus sensitivity analysis excluding TgAb-positive patients (n = 361).

| Predictor | Primary model (full cohort, n = 420) β | Primary model OR (95% CI) | P value | Sensitivity model (excluding TgAb-positive, n = 361) β | Sensitivity model OR (95% CI) | P value |
| --- | --- | --- | --- | --- | --- | --- |
| RS (per 1-unit increase in z-score) | 10.96 | 5.76×10⁴ (3.95×10³ – 8.39×10⁵) | <0.001 | 10.91 | 5.45×10⁴ (2.97×10³ – 1.00×10⁶) | <0.001 |
| Age (per 1-year increase) | 0.08 | 1.080 (1.010–1.160) | 0.021 | 0.09 | 1.090 (1.010–1.180) | 0.019 |
| Tumor diameter (per 1-cm increase) | 0.50 | 1.650 (1.190–2.290) | 0.003 | 0.57 | 1.770 (1.230–2.550) | 0.002 |
| Regional lymph-node metastasis (yes vs no) | 0.55 | 1.740 (0.700–4.320) | 0.235 | 0.54 | 1.710 (0.640–4.550) | 0.282 |
| Extrathyroidal extension (present vs absent) | −0.95 | 0.390 (0.150–0.980) | 0.044 | −0.95 | 0.390 (0.140–1.050) | 0.061 |

Abbreviations: OR, odds ratio; CI, confidence interval; RS, radiomics score; TgAb, anti-thyroglobulin antibody; RAI, radioactive iodine.
The dependent variable is unsatisfactory response to RAI at 6 months (1 = unsatisfactory, 0 = satisfactory).
